# Drug-resistant *Staphylococcus aureus* bacteria detection by combining surface-enhanced Raman spectroscopy (SERS) and deep learning techniques

**DOI:** 10.1038/s41598-021-97882-4

**Published:** 2021-09-16

**Authors:** Fatma Uysal Ciloglu, Abdullah Caliskan, Ayse Mine Saridag, Ibrahim Halil Kilic, Mahmut Tokmakci, Mehmet Kahraman, Omer Aydin

**Affiliations:** 1 Department of Biomedical Engineering, Erciyes University, 38039 Kayseri, Turkey; 2 IMaR Technology Gateway, Munster Technological University, Kerry, Ireland; 3 Department of Biomedical Engineering, Iskenderun Technical University, 31200 Hatay, Turkey; 4 Department of Chemistry, Gaziantep University, 27310 Gaziantep, Turkey; 5 Department of Biology, Gaziantep University, 27310 Gaziantep, Turkey; 6 ERNAM-Nanotechnology Research and Application Center, Erciyes University, 38039 Kayseri, Turkey; 7 ERKAM-Clinical Engineering Research and Application Center, Erciyes University, 38040 Kayseri, Turkey

**Keywords:** Analytical chemistry, Biomedical engineering, Nanobiotechnology

## Abstract

Over the past year, the world's attention has focused on combating COVID-19 disease, but the other threat waiting at the door—antimicrobial resistance should not be forgotten. Although making the diagnosis rapidly and accurately is crucial in preventing antibiotic resistance development, bacterial identification techniques include some challenging processes. To address this challenge, we proposed a deep neural network (DNN) that can discriminate antibiotic-resistant bacteria using surface-enhanced Raman spectroscopy (SERS). Stacked autoencoder (SAE)-based DNN was used for the rapid identification of methicillin-resistant *Staphylococcus aureus* (MRSA) and methicillin-sensitive *S. aureus* (MSSA) bacteria using a label-free SERS technique. The performance of the DNN was compared with traditional classifiers. Since the SERS technique provides high signal-to-noise ratio (SNR) data, some subtle differences were found between MRSA and MSSA in relative band intensities. SAE-based DNN can learn features from raw data and classify them with an accuracy of 97.66%. Moreover, the model discriminates bacteria with an *area under curve* (AUC) of 0.99. Compared to traditional classifiers, SAE-based DNN was found superior in accuracy and AUC values. The obtained results are also supported by statistical analysis. These results demonstrate that deep learning has great potential to characterize and detect antibiotic-resistant bacteria by using SERS spectral data.

## Introduction

Antimicrobial resistance is a growing problem globally, and 700,000 people die because of resistant infections annually. By 2050, it will threaten 10 million lives a year^1^. Inappropriate prescribing increases unnecessary antibiotic consumption, which triggers antimicrobial resistance with a short period^2^. Antibiotic resistance can be prevented by prescribing the proper antibiotics and raising public awareness. As another solution, new antibiotics can be discovered to compensate for antibiotic resistance. However, the number of discovered and approved antibiotics has declined between 1980 and 2014^3^. Hence, rapid and correct diagnosis of bacterial infections is required to prescribe the right antibiotic, and this is so crucial to curb antibiotic resistance.

Antimicrobial susceptibility test (AST), categorized as phenotypic and genotypic, is utilized to determine bacteria's antibiotic resistance. Phenotypic AST is reliable; however, it contains a time-consuming culturing step. On the other hand, genotypic AST provides fast results since it eliminates the need for culturing. Although it is highly sensitive, the existence of resistance genes does not mean expressed resistance. Further, genotypic AST requires trained personnel with advance knowledge^4^. Therefore, alternative diagnostic tools are needed for fast and reliable detection of antibiotic resistance.

Surface-enhanced Raman spectroscopy (SERS) is a promising biomedical diagnostic tool and spans broad applications in the biomedical field^5–9^. Within the last two decades, it has been successfully applied to discriminate bacteria as well^10–12^. Therefore, the SERS technique also has a significant potential to detect bacteria's antibiotic resistance^13,14^. Although SERS provides unique molecular information, SERS spectra of antibiotic-resistant and susceptible bacteria show subtle spectral differences. Therefore, the SERS technique requires advanced data processing algorithms to capture these minor differences. A vast majority of publications have reported that machine learning techniques can be employed to discriminate antibiotic-resistant and susceptible bacteria by using data obtained from SERS^15–18^.

There are three main steps, including preprocessing, feature extraction, and classification, to determine bacteria from the SERS data by using machine learning techniques. Therefore, obtaining a classification model is very tedious and time-consuming due to the rigid interdependency of the steps. Although some traditional machine learning techniques give reasonable accuracy results to detect the type of bacteria, they have several disadvantages, including overfitting, underfitting, requiring many user-supplied parameters, needing advanced nonlinear optimization techniques, etc. Fortunately, these challenges can be overcome using deep learning models whose achievement originates from large data volumes and sophisticated computational abilities. Deep learning models can learn significant raw data patterns without using advanced preprocessing and feature extraction techniques^19^. Thus, these algorithms seriously reduce the need for feature engineering^20^.

In recent years, deep learning algorithms have been applied to analyze spectroscopic signals^19^. However, the number of studies in spectral analysis with deep learning is limited^21–23^. A few studies have been reported to discriminate antibiotic-resistant and susceptible bacteria with deep learning algorithms using Raman spectroscopy and SERS^24,25^. Ho et al. have utilized a convolutional neural network (CNN) to classify 30 common bacterial pathogens data obtained from Raman spectroscopy^24^. They have also shown that CNN distinguished Methicillin-resistant *Staphylococcus aureus* (MRSA) and methicillin-susceptible *S. aureus* (MSSA) bacteria with 89 ± 0.1% accuracy using Raman spectral data. Raman spectroscopy has a low signal-to-noise ratio (SNR) due to the low scattering efficiency. This low SNR may be masked easily by background noise. Since noisy Raman spectra make it difficult to detect subtle differences between spectra, the performance of the classifier may be decreased. Thrift et al*.* reported that variational autoencoders discriminate *Escherichia coli* and *Pseudomonas aeruginosa* bacteria's metabolite profiles based on their SERS data^25^.

Among deep learning algorithms, autoencoders have been increasingly used in medical applications in recent years^26,27^. Although CNN is one of the most known and used deep learning algorithms, it is often preferred especially in the analysis of image data and provides successful results^28,29^. Therefore, to classify 1D SERS signals of MRSA and MSSA stacked autoencoder (SAE) based deep neural network (DNN) was preferred in this study. Autoencoders are used for dimensionality reduction from the original high-dimensional space to a new low-dimensional space. They are trained to reconstruct its input at the output by minimizing the loss between the original data and the data decoded from this representation. An autoencoder consists of two main parts: (1) encoder maps the input data into a low-dimensional feature space and (2) decoder trained to reconstruct the input from the features extracted by the encoder^19^. Becasuse of nonlinear activation functions, autoencoders can learn complex hierarchical features. To extract more complex new features, autoencoders can be stacked combining the desired number of trained autoencoder encoder parts and thus, an SAE is obtained. After the training of SAE in an unsupervised fashion, a powerful automatic feature extractor is acquired. A DNN is formed by combining obtained SAE and a softmax classifier that performs classification^30^. Thus, SAE-based DNN is formed to discriminate MRSA and MSSA spectral data.

Herein, we use an SAE-based DNN to classify MRSA and MSSA bacteria's SERS spectra. MRSA has been shown as a serious threat according to the 2019 report on antibiotic resistance threats in the United States^31^. These bacteria are resistant to β-lactam antibiotics, including penicillin, cephalosporin, and carbapenem^32^. Undoubtedly, rapid and accurate detection of antibiotic resistance profiles of *S. aureus* bacteria will both reduce morbidity and mortality and slow down the development of antibiotic resistance.

We hypothesized that the cell wall structure of MRSA and MSSA might show some differences due to the resistance mechanism. SERS able to reflect these differences at the collected spectra. The discrimination of subtle spectral differences between MRSA and MSSA is a challenging problem. This study addresses this challenge by using an SAE-based DNN to discriminate antibiotic-resistant and susceptible bacteria.

**Figure 1:**
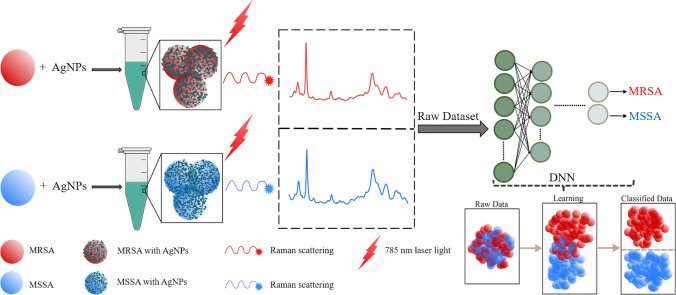
General workflow of deep learning-based spectral data analysis for the discrimination of antibiotic-resistant bacteria.

Figure 1 illustrates the general workflow of this study. We first collected SERS spectra of MRSA and MSSA using silver nanoparticles (AgNPs) as SERS substrate. The raw spectral dataset of antibiotic-resistant and susceptible *S. aureus* bacteria was classified using SAE-based DNN. Further, some traditional machine learning algorithms such as support vector machine (SVM), linear discriminant analysis (LDA), k-nearest neighbors (KNN), decision tree (DT), and a neural network (NN) were used to compare the performances of the DNN and traditional classifiers. To the best of our knowledge, this is the first report for discrimination of MRSA and MSSA SERS data by using SAE-based DNN. This work shows that SERS together SAE-based DNN can successfully discriminate MRSA and MSSA bacteria.

## Materials and methods

### AgNPs synthesis

AgNPs were synthesized according to the method reported by Lee and Meisel^33^. Briefly, 90 mg silver nitrate (AgNO_3_, Merck, Darmstadt, Germany) was dissolved in 500 mL distilled water. This solution was heated until boiling. Then, 10 mL aliquot of 1% sodium citrate (Merck, Darmstadt, Germany) was added drop by drop into the solution. The solution was kept boiling for about 1 h. Synthesized AgNPs were centrifuged at 5500 rpm for 1 h and discard a portion of the supernatant to form 4 × concentrated AgNPs. To characterize AgNPs, the absorption spectrum was measured in the range of 300–700 nm using Thermo Scientific Evolution 201 UV–Vis spectrophotometer (Waltham, USA). The morphology of the nanoparticles was determined using Scanning Transmission Electron Microscope (STEM) imaging (Zeiss GeminiSEM) that is performed with an acceleration voltage of 25 kV.

### Bacterial sample preparation

*Staphylococcus aureus* strains (MRSA and MSSA) were used in this study. 19 MRSA and 1 MSSA bacteria were obtained from our microorganism collection (Gaziantep University, Biology Department) with the ethical permission of Gaziantep University clinical research ethics committee (09.06.2014/195). In addition, *S. aureus* type strains ATCC 6538, and ATCC 25923 were purchased from The American Type Culture Collection. The antibiotic resistance of MRSA and MSSA was confirmed by the presence or absence of *mec A* gene using the Polymerase Chain Reaction (PCR) technique. Furthermore, the disc diffusion method was also used for the antibiotic resistance confirmation, and details can be found in our previous study^15^. According to PCR and disc diffusion method results, methicillin resistance was found in all MRSA bacteria.

The bacteria were grown at 37 °C on Mueller Hinton agar (Merck, Darmstadt, Germany). The samples were gathered with sterile inoculating loops after the 24 h cultivation. The collected bacteria were added into 1 mL ultrapure water, quickly vortexed, and centrifuged for 5 min at 7500 rpm. The supernatant was discarded, and this procedure was repeated 3 times. A 5 µL aliquot of each washed bacterial sample was added into 100 µL 4 × concentrated AgNPs colloidal suspension. Then, samples were shortly vortexed to form homogenous mixtures. A 5 µL of each mixture was immediately dropped on CaF_2_ slide and dried at room temperature about 30 min for SERS measurements.

### SERS measurements

SERS measurements were performed using Renishaw inVia Reflex Raman Microscopy System (Renishaw plc., Wotton -under-Edge, UK) using 50x (0.75 NA) microscope objective with 1 s exposure from 785 nm excitation under the ~ 3 mW laser power. The laser spot size was calculated as 1.3 µm (1.22 × λ/NA). The spectra were collected with a 5 µm step size to prevent overlapping. Two datasets were acquired on different days for reproducibility. In the first data set, spectra were collected from 4 different regions of the samples prepared for each isolate. Under the given measurement parameters, 750–775 spectra were collected from each isolate within 20 min. Samples were also collected from the second data set in the same way. A total of 1500–1550 spectra were collected from each isolate from two dataset on different days. Hence, the total dataset consisted of 33,975 spectra and this large dataset were acquired about 15 h. 1200 lines/mm^−1^ grating was used, providing a spectral range from 550 to 1700 cm^−1^.

### Outlier detection

To detect outliers in the dataset, the isolation forest algorithm proposed by Liu et al. was utilized^34,35^. This algorithm was applied in R programming language v.3.6.343^36^ using isolationForest() function in the solitude package^37^. In this technique, anomalies are detected by isolating samples in the dataset. To isolate samples, Isolation Tree or iTree a binary tree structure is used. Anomalies are isolated closer to the root of an iTree due to the susceptibility to isolation. On the other hand, normal samples are isolated at the deeper end of an iTree. Isolation forest or iForest algorithm constructs an ensemble of iTrees. In a given dataset, anomalies have short average path length on the iTrees. Thus, a lower average path length for a sample indicates a higher likelihood that the sample is an anomaly. The average path length *c(n)* is calculated for a given dataset of *n* instances as following^35^:

1 $$c\left( n \right) = 2H\left( {n - 1} \right) - \left( {\frac{{2\left( {n - 1} \right)}}{n}} \right)$$

where *H(i)* is the harmonic number estimated by the Euler’s constant. The anomaly score *s* for a given sample *x* is defined as below:

2 $$s\left(x,n\right)={2}^{-\frac{E(h\left(x\right))}{c(n)}}$$

where *h(x)* is the path length of *x*, *E(h(x))* is the average of *h(x).* In Eq. (2):

3 $$\begin{array}{*{20}l} {} \hfill & {E\left( {h\left( x \right)} \right) \to c\left( n \right), s \to 0.5} \hfill \\ {{\text{When}}} \hfill & {E\left( {h\left( x \right)} \right) \to 0,s \to 1} \hfill \\ {} \hfill & {E\left( {h\left( x \right)} \right) \to n - 1, s \to 0} \hfill \\ \end{array}$$

It is decided by looking at these score values whether the spectrum in the dataset is an anomaly or not. According to Eq. (3), using the anomaly score *s* following assessments can be made: (a) the score values close to 1 indicate that those points are definitely anomalies, (b) the score values less than 0.5 indicates that they are not anomalies, and (c) if all the score values are around 0.5 indicates that the whole data do not have any anomaly^34^. Moreover, spectra with score values greater than 0.7 are accepted anomaly^34^ and discarded from the data set.

### SAE-based DNN

SAE-based DNN presented here consists of the encoder layers of trained autoencoders and a softmax classifier.

Autoencoders aim to reduce high-level features to a simpler representation in a low-dimensional space. They can extract better features than hand-engineered features since they can learn complex hierarchical features from the data. Although mean, standard deviation, and relative intensities of some band positions in the spectra, etc., are generally used in hand engineered-feature extraction, some features that will technically correspond to them but are not completely related to them are extracted in autoencoders. Features extracted from the autoencoder do not have a pattern; however, they are related to statistical properties of the input data such as the mean, standard deviation of the signal, or the determination of hard transitions in the signal.

A single autoencoder consists of encoder and decoder parts. The encoder part takes the input vector *x* (*x* ϵ R^M×1^) and maps this vector into hidden representation *c* known as code^38^. This process is as follows:

4 $$c_{i} = f\left( {b_{i1} + W_{i1} x} \right)$$

where *c*_*i*_ ϵ R^M×1^ is the code, f is the encoding function, *b*_*i1*_ ϵ R^M×1^ is the bias vector, and *W*_*i1*_ ϵ R^M×N^ is the weight matrix of the encoder. The encoder part of an autoencoder is trained using unsupervised fashion to dig significant feature information.

The decoder part reconstructs the input vector as *x̂*. Thus, an autoencoder tries to generate its input at the output layer by minimizing the error as much as possible between input *x* and output *x̂*. Decoding of *c*_*i*_ is expressed as follows:

5 $$\hat{x} = g\left( {b_{i2} + W_{i2} c_{i} } \right)$$

where *g* is the encoding function, *b*_*i2*_ ∈ R^N×1^ is the bias vector, and *W*_*i2*_ ∈ R^N×M^ is the weight matrix of the decoder.

The objective function minimizing the error between the input and output is expressed:

6 $$J\left( {W_{i} ,b_{i} ,x_{i} } \right) = \frac{1}{2}\left\| {h_{{Wi,bi}} \left( {x_{i} } \right) - x_{i} } \right\|^{2}$$

Two regularization terms are added to Eq. (6), as seen in Eq. (7). *λ* is a regularization term and is used to prevent overfitting. *β* is the weight of the sparsity penalty term and is used to allow the autoencoder discovering hidden features related to raw data. The term *ρ* is the constant sparsity parameter, and *ρ̂*_*j*_ is the mean activation value of the *j*th neuron in the hidden layer.

7 $$J = \frac{1}{2}\left\| {h_{Wi,bi} \left( {x_{i} } \right) - x_{i} } \right\|^{2} + \lambda \left( {\left\| {W_{i} } \right\|_{2}^{2} } \right) + \beta \mathop \sum \limits_{j = 1}^{M} KL\left( {\left. \rho \right\|\rho_{j} } \right)$$

*KL(ρ‖ρ̂*_*j*_) term expresses the Kullback–Leibler divergence:

8 $$KL(\left. \rho \right\|\hat{\rho }_{j} ) = \rho log\frac{\rho }{{\hat{\rho }}} + (1 + \hat{\rho }_{j} )log\frac{{1 - \rho }}{{1 - \hat{\rho }_{j} }}$$

The number of neurons at the hidden layer is generally chosen lower than the input layer size. Therefore, the autoencoder is forced to extract new features with an unsupervised approach.

Softmax classifier, which generalizes logistic regression, is the supervised layer of the deep learning model^38^. It is based on the softmax function and used to classify the learned features by the autoencoder. The softmax classifier's cost function attempts to decrease the difference between the actual label value and model output by tuning the model parameters.

**Figure 2:**
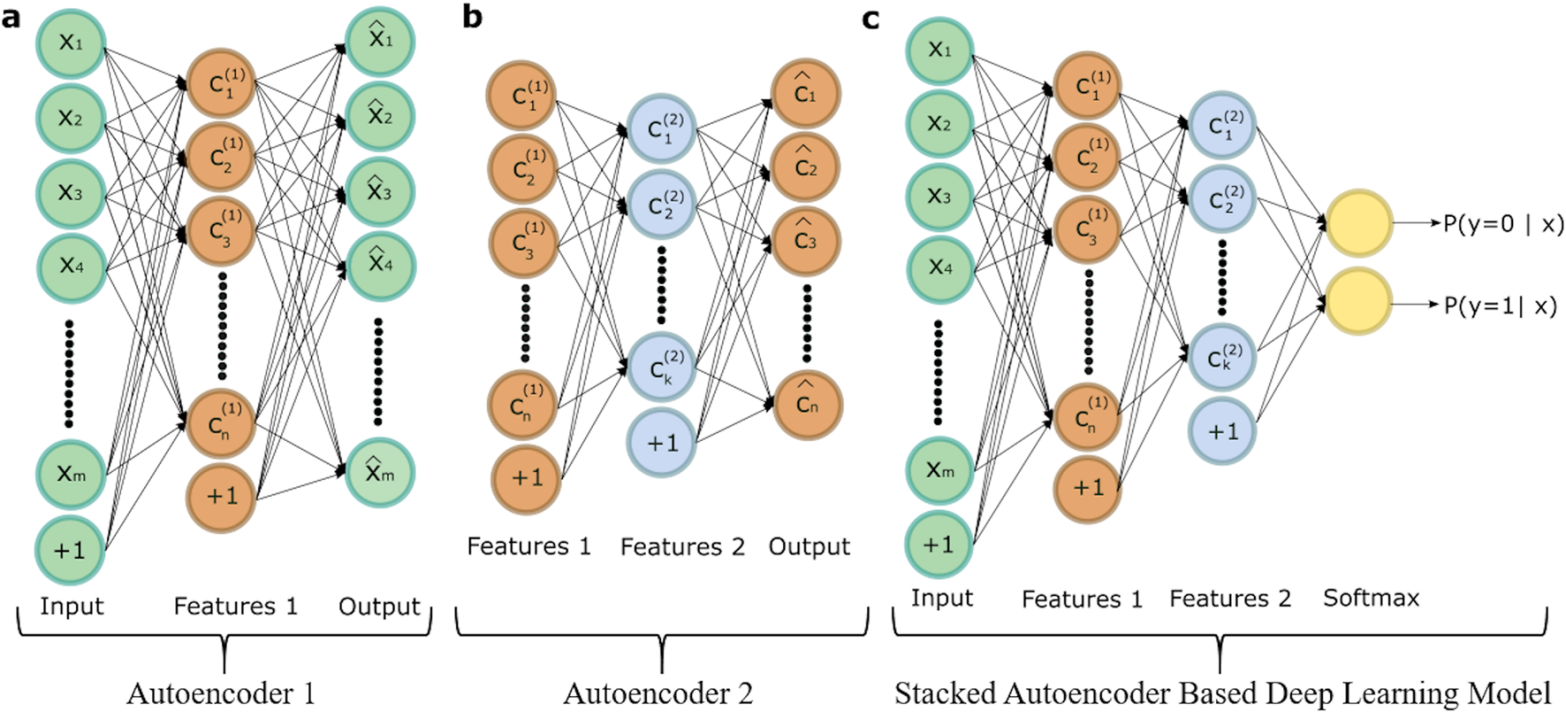
The structure of the proposed deep learning model. (**a**) First autoencoder that reveals the features from the input data. (**b**) Second autoencoder that reveal the new features from the first autoencoder's hidden layer. (**c**) The deep learning model that is formed stacking the two autoencoders and softmax classifier.

To form an SAE-based DNN, desired number of the encoder part of the trained autoencoders and softmax classifier are joined together. The performance of the SAE-based DNN can be increased by performing backpropagation on the model. This procedure is known as fine tuning and significantly improved the results of the SAE. Figure 2 shows frameworks of the SAE-based DNN used in this study. The training procedure of the DNN is as follows: (a) The first autoencoder is trained by feeding a 1024-dimensional input vector to a hidden layer with 30 neurons, as illustrated in Fig. 2a, (b) the second autoencoder is trained to feed the first autoencoder's hidden layer to the second autoencoder's hidden layer with 15 neurons as depicted in Fig. 2b, (c) the encoder parts of the trained autoencoders and softmax layer are consecutively connected to construct an SAE-based deep learning model, as seen in Fig. 2c, and (d) finally, to achieve fine-tuning the backpropagation algorithm is performed, and weights are updated with labeled training data.

One of the main challenges in DNN models is hyperparameter optimization. It is a difficult and time-consuming process. The amount of data needed in such models should be much larger than the number of hyperparameters in the network. Otherwise, over-fitting an undesirable situation occurs. In such a scenario, while the network produces very successful results on the training set, it cannot generalize the model on the unseen test set. Therefore, hyperparameter optimization and the number of samples in the dataset is critically important. The hyperparameter optimization was done by random search. The specific parameters used to construct SAE-based DNN are given in Supplemental Table S1. Ten-fold cross-validation was performed to prevent overfitting, to provide fair classification results. SAE-based deep learning model was constructed with MATLAB software (The MathWorks, Natick, USA).

To compare the performance of SAE-based DNN with the state-of-the-art classifiers, SVM, LDA, KNN, DT, and NN were used. These classifiers were performed using MATLAB software. The whole data were standardized before applying to SAE-based DNN and traditional classifiers. In standardization procedure all spectral vectors were standardized using standard normal variate. In the standardization step the mean value and standard deviation of each spectral vector were set to 0 and 1, respectively. Then, all standardized vectors were scaled in the range of [0, 1]. Here, 1 corresponds to the maximum value of the features in the standardized data set. Thus, each data point is scaled into an interval suitable to the logistic sigmoid activation function that is used encoder and decoder parts of the autoencoder.

### Statistical analysis

The Mann Whitney U test was utilized for statistical analysis. This analysis was performed using MATLAB software and > 95% confidence level was selected (*P* < 0.05 means there is a significant difference between the groups).

## Results and discussion

### SERS spectra of MRSA and MSSA

There are a lot of SERS substrates using in the studies. These substrates can be different forms such as colloidal, solid, and flexible^39^. Noble metal colloids^40–44^ and noble metal surfaces^17^ are broadly utilized in bacteria detection studies. Among them, solid substrates provide good repeatability, but they have low SNR ratio and are not easy to manufacture^45^. Bacteria and nanoparticles directly interact in many points when noble metal colloids are used as the SERS substrate, and this situation increases the SNR of the collected spectrum. However, reproducibility of the collected spectra can be low because nanoparticles do not properly form hot-spots. To improve reproducibility, concentrated nanoparticles are used to increase possibility of hot-spot formation^10^. Gold (Au) and silver (Ag) are mainly used due to the higher enhancement factor in SERS^39^. Between them Ag are widely preferred in bacterial SERS studies since AgNPs provides high signal enhancement factor, wide tunability, and are cost effective^43,46^. Therefore, the citrate reduced AgNPs were used in this study due to their high SERS activity and providing reproducible spectra. The UV–Vis spectrum and STEM image of the synthesized AgNPs are showed in Supplemental Fig. S1. The maximum absorption of AgNPs was found at 420 nm, and they were mostly spherical in the range of 50–60 nm as seen in the inserted image in Supplemental Fig. S1.

**Figure 3:**
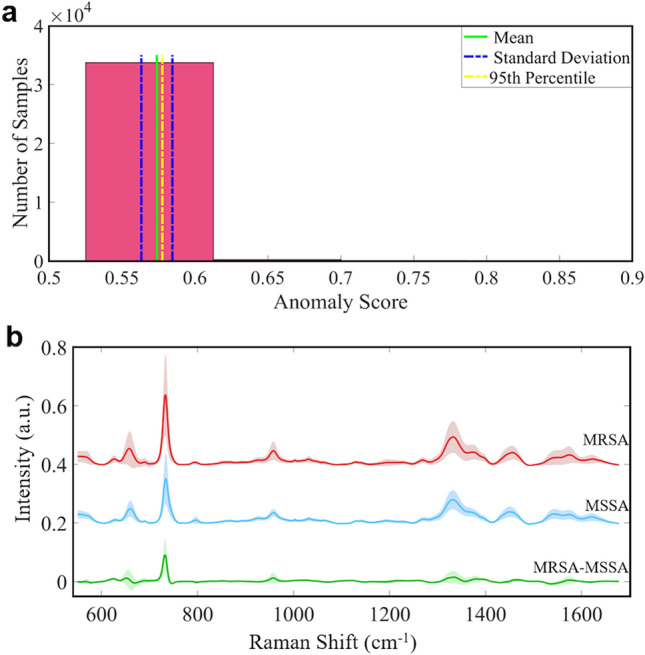
(**a**) Histogram plot of the anomaly score values obtained by the isolation forest algorithm. (**b**) Normalized average SERS spectra ± standard deviation of MRSA, MSSA and the difference of MRSA-MSSA.

To collect a large dataset 33,975 spectra were acquired from 19 MRSA and 3 MSSA bacterial isolates for 2 measurement times. The isolation forest algorithm was used to determine whether there is an outlier in the data. The results of this algorithm are shown in Fig. 3a. The score values that show whether the spectrum is outlier or not are in the range of [0.572, 0.875]. The mean ± standard deviation of them was found to be 0.574 ± 0.009. Further, the 95th percentile of the score values was found as 0.577; in other words, 95% of the score values are lower than 0.577. A vast amount of score values are distributed around 0.5, as seen in Fig. 3a indicates that these spectra do not really anomaly. Only 24 score values were determined greater than 0.7, and the spectra to which these values belong were accepted as anomalies and removed from the dataset. Different score values belong to a few spectra are depicted in Supplemental Fig. S2. As illustrated in this figure score values above 0.7 are seen differently from other spectra.

SERS spectra are acquired by illuminating the whole bacterium, which interacts with the colloidal AgNPs. Thus, the collected spectra are generated by the outermost of the bacterial cell wall because of the distance dependence of SERS enhancement^47,48^. Since the SERS spectra collected from bacteria reflect composition of the cell wall in close proximity with the SERS substrate. Peptidoglycan layer, teichoic acids, surface proteins, capsular polysaccharides, and phospholipids are the primary components of the bacterial cell wall^49^. The peptidoglycan layer in the bacterial cell wall is a protective envelope found on the outside of the cytoplasmic membrane where composes of glycan strands crosslinked with short peptides^50^. *Staphylococcus aureus* which is a gram-positive bacterium has a thick peptidoglycan layer at the outermost of the cell wall. Peptidoglycan biosynthesis is an excellent target for most of the antibiotics, including β-lactams^49^. Correspondingly, some structural differences are anticipated between MRSA which is resistant to β-lactams and MSSA cell wall that SERS could reveal these differences.

The normalized mean SERS spectra ± standard deviation of MRSA and MSSA are depicted in Fig. 3b in the range of 550–1700 cm^−1^. The shaded area shows the variations of measured spectral intensities. SERS spectra of MRSA and MSSA bacteria depict a lot of similar peak positions except for some differences in relative band intensities. The primary SERS spectra of MRSA and MSSA are characterized by strong bands at 658 cm^−1^ (COO−deformation of guanine)^51^, 732 cm^−1^ (flavin adenine dinucleotide derivatives, glycosidic ring mode of the N-acetyl D-glucosamine and N-acetylmuramic)^52–54^, 958 cm^−1^ (CN deformation of saturated lipids)^42^, 1333 cm^−1^ (C-N stretching mode of Adenine)^55^, 1450 cm^−1^ (CH_2_ deformation of saturated lipids)^56^, and 1576 cm^−1^ (CN stretching of amide II)^57^. It is clearly illustrated in Fig. 3b that the spectral profile of MRSA and MSSA bacteria is quite similar. However, a notable difference between MRSA and MSSA is the intensity of 732 cm^−1^ peak position. Interestingly, this sharp peak is drastically increased in MRSA. The source of this band is explicitly assigned by some groups to flavin adenine dinucleotide (FAD) derivatives and glycosidic ring mode of the N-acetyl D-glucosamine (NAG) and N-acetylmuramic (NAM) which are building blocks of the peptidoglycan layer^52–54^. Kahraman et al. reported that both band assignments are correct, and it is possible for bands originating from both NAG and FAD to overlap^58^.

The prominent increase in the 732 cm^−1^ band in MRSA may indicate differentiation in the peptidoglycan layer of MRSA. Since β-lactam antibiotics work by targeting Penicillin Binding Proteins (PBPs) in the peptidoglycan layer. It is possible to observe some differences for peak intensities or positions originating the peptidoglycan layer. Genotypic changes that cause antibiotic resistance are usually represented in the induced phenotype that inhibits the action of an antibiotic. Garcia et al. measured the cell wall and septum thickness of MRSA and MSSA^59^. They report that the cell wall and septum thickness of MRSA and MSSA have been found statistically different. Besides, they have correlated the cell wall thickness of MRSA with the resistance mechanism. There are also some minor differences in the intensity of 658, 958, and 1333 cm^−1^ peaks. These peaks are more intense for MRSA than MSSA. The changes between MRSA and MSSA SERS spectra indicate that there is a variation in the amount of some biomolecules in the cell wall. Thus, SERS has the potential to reveal the variations between MRSA and MSSA.

### SAE-based DNN and traditional classifiers for the classification of MRSA and MSSA SERS spectra

The spectral features of MRSA and MSSA are highly similar, as clearly seen in Fig. 3b. There are only some subtle differences in the relative intensities of the SERS peaks of MRSA and MSSA that can be determined by the naked eye such as 1500–1600 cm^−1^ spectral region as seen in Fig. 3b. However, trained personnel is needed to determine this difference for every spectrum. Furthermore, this difference may not be seen in some spectra collected from different isolates or it may not be prominent as seen in Fig. 3b. This situation necessitates using a robust algorithm for data analysis. To correctly classify SERS spectral data of MRSA and MSSA bacteria, an SAE-based DNN was utilized.

The total dataset consists of 33,951 SERS spectra of MRSA and MSSA. The 29,452 of them belong to 19 MRSA isolates, and the remaining belong to 3 MSSA isolates. The entire data were used without preprocessing and feature extraction steps. In spectral data analysis, preprocessing and feature extraction are two important steps that show a major impact on the classifier performance. However, misuse of these methods can seriously distort the original data and adversely affect classifier performance^60^.

**Figure 4:**
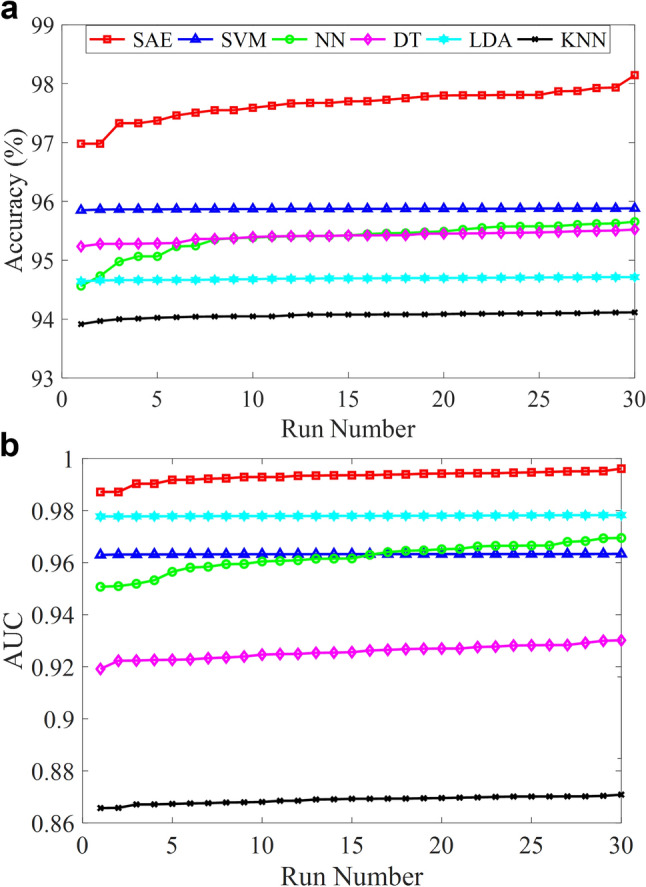
Performance comparisons of SAE-based DNN and traditional classifiers. (**a**) Accuracies of classifiers for 30 runs. (**b**) AUC values obtained from ROC curve of classifiers for 30 runs.

In this study, preprocessing steps such as noise elimination were not required since SERS can provide high SNR data. Moreover, feature extraction, which is a challenging process, was not used due to the ability of SAE-based DNN about revealing critical features from the raw data. This deep learning model can extract relevant features thanks to the multiple autoencoders. Thus, the dimension of the input data passing through the hidden layers of each autoencoder is significantly reduced. The raw input data were standardized before applying SAE-based DNN and traditional classifiers. The whole raw data were shuffled randomly before implementing into classifiers. Ten-fold cross-validation technique was used to measure the performance of the model and this procedure was repeated for 30 runs for each classifier. The mean accuracies of SAE-based DNN and traditional classifiers for 30 runs are depicted in Fig. 4a. The accuracy values seen in Fig. 4a are sorted in ascending order to illustrate the accuracy values between the classifiers in depth. Therefore, the accuracy value obtained at the 30th run is seen as the highest value. It is clearly seen that SAE based deep learning model shows better classification performance than traditional classifiers. This model provides the best mean accuracy with 97.66 ± 0.26%, among others. Traditional classifiers have close classification performance and SVM gives slightly better results with 95.87 ± 0.01%, among them. The mean, maximum, minimum, and standard deviation of each classifier accuracies acquired from 30 runs are given in Table 1.

**Table 1:** Mean accuracies of the classifiers with maximum, minimum and standard deviation for 30 runs.

Method	Mean (%)	Maximum (%)	Minimum (%)	Standard deviation
SAE	97.66	98.14	96.98	0.26
SVM	95.87	95.88	95.85	0.01
NN	95.38	95.66	94.56	0.26
DT	95.41	95.52	95.23	0.08
LDA	94.69	94.71	94.64	0.02
KNN	94.06	94.12	93.91	0.05

**Figure 5:**
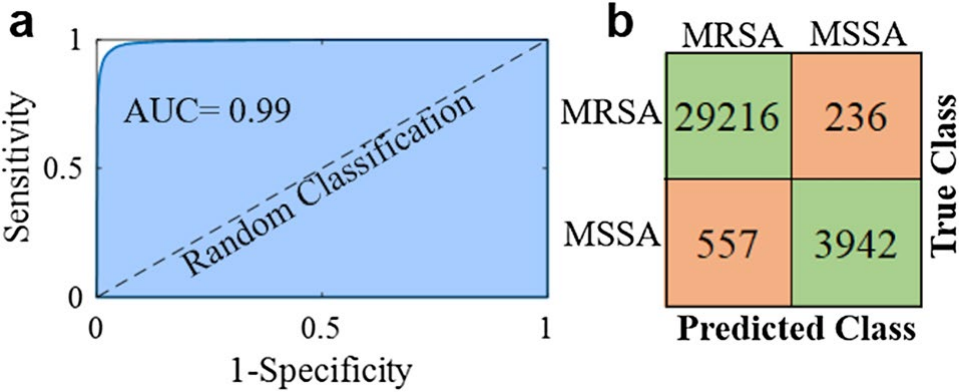
Binary classification results of MRSA and MSSA by the SAE based deep learning model. (**a**) The ROC curve with an AUC of 0.99. (**b**) Confusion matrix showing the results of ten-fold cross validated bacterial identification.

A classifier performance can be measured by different metrics, and accuracy is one of them. However, accuracy is not enough to measure the performance of a classifier. Especially in dataset where the amount of data in classes is unbalanced, measuring the classifier performance with the only accuracy parameter does not give reliable results. The receiver operating characteristic (ROC) curve shows a classifier performance for all classification threshold values. This curve is plotted with the true positive rate (y-axis) against the false positive rate (x-axis). The area under a ROC curve abbreviated as AUC is frequently used to measure classification performance and is one of the most important evaluation techniques. The value of AUC is in the range of [0, 1] and when the AUC value is getting closer to 1, classification error decreases. Figure 4b illustrates the AUC values of SAE-based DNN and traditional classifiers for 30 runs. As seen in Fig. 4b, SAE based deep neural network has the best AUC values through 30 runs. The mean AUC value of it was found 0.993 ± 0.002 which means the deep learning model can distinguish MRSA and MSSA with a high performance as depicted in Fig. 5a. KNN gives the worst AUC values for each run, while LDA, SVM, NN, and DT have better results than traditional classifiers. In addition, the mean, maximum, minimum, and standard deviation of each classifier AUC values obtained from 30 runs are provided in Table 2. The confusion matrix of SAE-based DNN is demonstrated in Fig. 5b. The accuracy, sensitivity, specificity, and precision of the deep learning model were calculated as 97.7%, 99.2%, 87.6%, and 98.2%, respectively. Misdiagnosing MRSA as MSSA causes more serious results than the reverse situation. Only 236 SERS spectra of MRSA were misdiagnosed as MSSA as seen in Fig. 5b.

**Table 2:** Mean AUC values of the classifiers with maximum, minimum and standard deviation for 30 runs.

Method	Mean	Maximum	Minimum	Standard deviation
SAE	0.9931	0.9961	0.9872	0.0020
SVM	0.9632	0.9634	0.9630	0.0001
NN	0.962	0.9695	0.9507	0.0054
DT	0.9257	0.9302	0.9192	0.0026
LDA	0.9780	0.9783	0.9777	0.0002
KNN	0.8688	0.8709	0.8657	0.0014

The above results show that SAE-based DNN has better classification performance than traditional classifiers. However, these findings should be supported with statistical analysis. Statistical analysis was used to compare the AUC values obtained from SAE-based DNN against state-of-the-art classification techniques such as SVM, NN, DT, LDA, and KNN for 30 runs. The Mann Whitney U test with a significance level of 0.05 was used for this purpose. The statistical results were interpreted according to the *p-values*. SAE-based DNN is found better than traditional classifiers in terms of the statistical analysis results for the discrimination of MRSA and MSSA spectral data.

The SAE-based DNN more accurately classified SERS spectral data of MRSA and MSSA bacteria. Our model applied here for rapid and reliable identification of antibiotic-resistant, and susceptible bacteria requires minimum sample preparation procedure, does not require special labels, and eliminates long incubation times required for phenotypic AST. Although raw data were used in our study, high classification accuracy and AUC were found thanks to the SERS technique with high SNR using SAE-based DNN which successfully extracts features from the data. Our group previously has applied traditional classifiers such as KNN, SVM, DT and naïve Bayes (NB) for the discrimination of MRSA, MSSA, and *Legionella pneumophila* bacteria^15^. KNN classifier has provided the best accuracy with 97.8%, among other techniques. However, as the size of the data set grows, the success of traditional classifiers falls behind the deep learning algorithms. Therefore, SAE-based DNN can provide more successful results for SERS spectral data of antibiotic-resistant bacteria with high accuracy and sensitivity.

Culture-based techniques are accepted as the gold standard for bacterial identification and antibiotic susceptibility test in clinical use. In this study, true classes in the confusion matrix were determined using both culture-based technique and PCR. Model predictions were then compared to results found at culture-based and PCR techniques that are accepted true classes. When the predictions of model largely overlap with the real class labels, the accuracy, sensitivity, specificity, and AUC values are high. As a result, the closer the model's accuracy value is to 100%, the more consistent the results are with clinical results. It is very important for the clinical use potential of the proposed method that the subtle differences in the SERS spectra of MRSA and MSSA can be distinguished by the model with a classification accuracy above 95%.

Although deep learning-based algorithms use sophisticated computing tools, thanks to the developing technology, these models can be used by people who are not experts in the field. It can be made available to people who need it using transfer learning. By transfer learning, the proposed method can be applied to similar problems. Thus, non-professionals about deep learning algorithms can be enabled to analyze the data using pre-trained networks.

**Figure 6:**
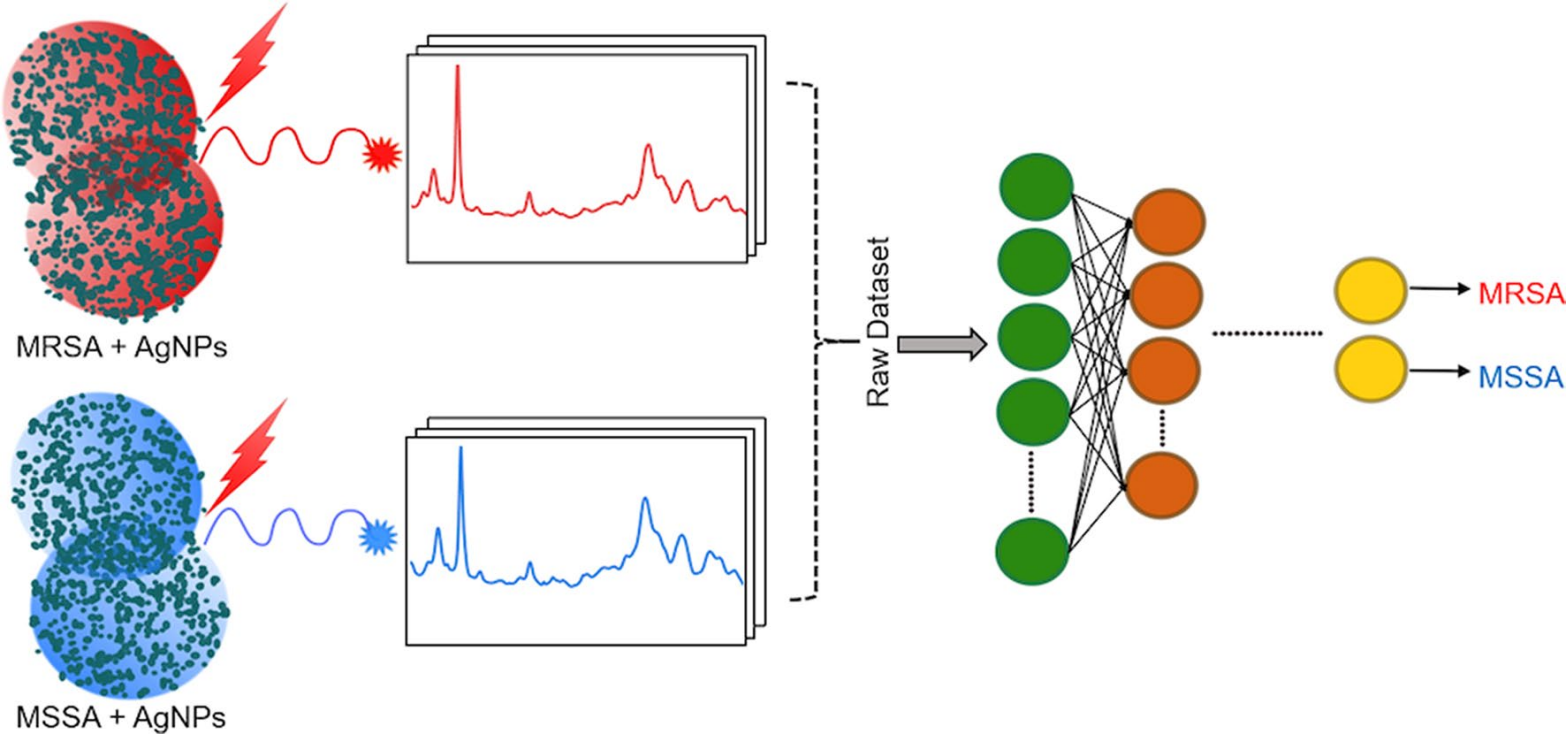
The graphical abstract of the study representing the main steps of the study consisting of AgNPs are mixed seperately with MRSA and MSSA and obtaining SERS spectra which are processed with deep learning techniques to distinguish the differences.

In this study, SERS spectral data of MRSA and MSSA bacteria have been successfully characterized and identified by SAE-based DNN. The results show that the proposed technique has a potential application for the detection of antibiotic-resistant bacteria in clinical utilization (Fig. 6). Compared with the phenotypic or genotypic AST techniques frequently used, the proposed method has advantageous in terms of easy use and fast detection. Thanks to the easy sample preparation and fast signal acquisition in SERS technique, antibiotic resistance in bacteria can be detected faster than culture-based techniques. Since culture based techniques requires additional about 24 h for antibiogram tests after the bacteria are grown in culture. Proposed method may render possible the detection of antibiotic resistant and susceptible strains in a shorter time. As a result, by reducing the unnecessary use of antibiotics, the development of antibiotic resistance will be slowed down, and morbidity and mortality will decrease.

## Conclusions

Rapid bacterial diagnosis is essential to combat antibiotic resistance. Label-free SERS provides a fingerprint spectrum of the sample with high SNR. Therefore, it is an attractive technique for bacterial identification studies. However, interpreting of SERS spectra is a difficult process due to the high molecular similarities of bacterial species. Detection of spectral differences between antibiotic-resistant and susceptible bacteria becomes even more difficult. Advanced data analysis techniques are indispensable at this step. Deep learning algorithms perform outstanding success by using SERS data for the discrimination of antibiotic-resistant bacteria.

Here we illustrate that SAE-based DNN can be used for the SERS-based label-free identification of antibiotic-resistant and susceptible strains of *S. aureus* bacteria. SERS technique providing high SNR reveals the subtle spectral differences between MRSA and MSSA. SAE-based DNN automatically extracts features needed for classification from the raw spectral data. Therefore, complex preprocessing and feature extraction steps are eliminated. Compared with the traditional classifiers, SAE-based DNN shows a more accurate diagnostic model with accuracy and AUC of 97.66%, 0.99, respectively. The proposed method provides a label free, rapid, and reliable technique with high sensitivity.

In conclusion, the proposed method has a great potential for clinical use, considering that rapid diagnostic methods have a great effect on combating antibiotic resistance. Further, this technique has a high application potential not only in the detection of antibiotic-resistant bacteria but also for a lot of label-free SERS applications in the biomedical field.

## Supplementary Information

Supplementary Information.

## Data Availability

The datasets generated during and/or analyzed during the current study are available from the corresponding author on reasonable request.
