# Supplementary material for: Drug-resistant Staphylococcus aureus bacteria detection by combining surface-enhanced Raman spectroscopy (SERS) and deep learning techniques
Source: Sci Rep. 2021 Sep 16;11:18444. doi: 10.1038/s41598-021-97882-4 (PMC8446005; doi:10.1038/s41598-021-97882-4)
Supplement: Supplementary file 1 — Supplementary Information. [file 41598_2021_97882_MOESM1_ESM.docx]

**Supplementary Information**

**Drug-resistant *Staphylococcus aureus* bacteria detection by combining surface-enhanced Raman spectroscopy (SERS) and deep learning techniques**

Fatma Uysal Ciloglu^1^, Abdullah Caliskan^2,3^, Ayse Mine Saridag^4^, Ibrahim Halil Kilic^5^, Mahmut Tokmakci^1^, Mehmet Kahraman^4*^, Omer Aydin^1,6,7*^

^1^ Department of Biomedical Engineering, Erciyes University, Kayseri, 38039, Turkey

email: [biomer@umich.edu](mailto:biomer@umich.edu) ; Tel: +90-352-207-6666/32984

^2^ IMaR Technology Gateway, Munster Technological University, Kerry, Ireland

^3^Department of Biomedical Engineering, Iskenderun Technical University, Hatay, 31200, Turkey

^4^ Department of Chemistry, Gaziantep University, Gaziantep, 27310, Turkey

email: [mskahraman46@gmail.com](mailto:mskahraman46@gmail.com) ; Tel: +90-342-317-1940

^5^ Department of Biology, Gaziantep University, Gaziantep, 27310, Turkey

^6^ ERNAM-Nanotechnology Research and Application Center, Erciyes University, Kayseri, 38039, Turkey

^7^ ERKAM-Clinical Engineering Research and Application Center, Erciyes University, Kayseri, 38040, Turkey

**Supplemental Table S1.** The specific parameters of the SAE-based DNN

| **Part of the SAE** | **# of neuron** | **# of max iteration** | **λ** | **β** | ***ρ*** |
| --- | --- | --- | --- | --- | --- |
| Autoencoder 1 | 30 | 400 | 0.004 | 2 | 0.42 |
| Autoencoder 2 | 15 | 400 | 0.001 | 1 | 0.02 |
| Softmax | - | 400 | 0.003 | - | - |


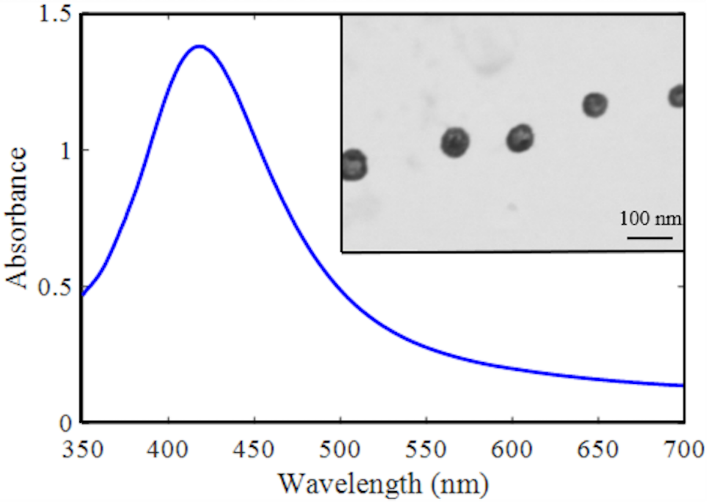


**Supplemental Figure S1.** The UV/Vis spectroscopy of the AgNPs. The inserted image shows the Scanning Transmission Electron Microscope (STEM) image of the AgNPs.


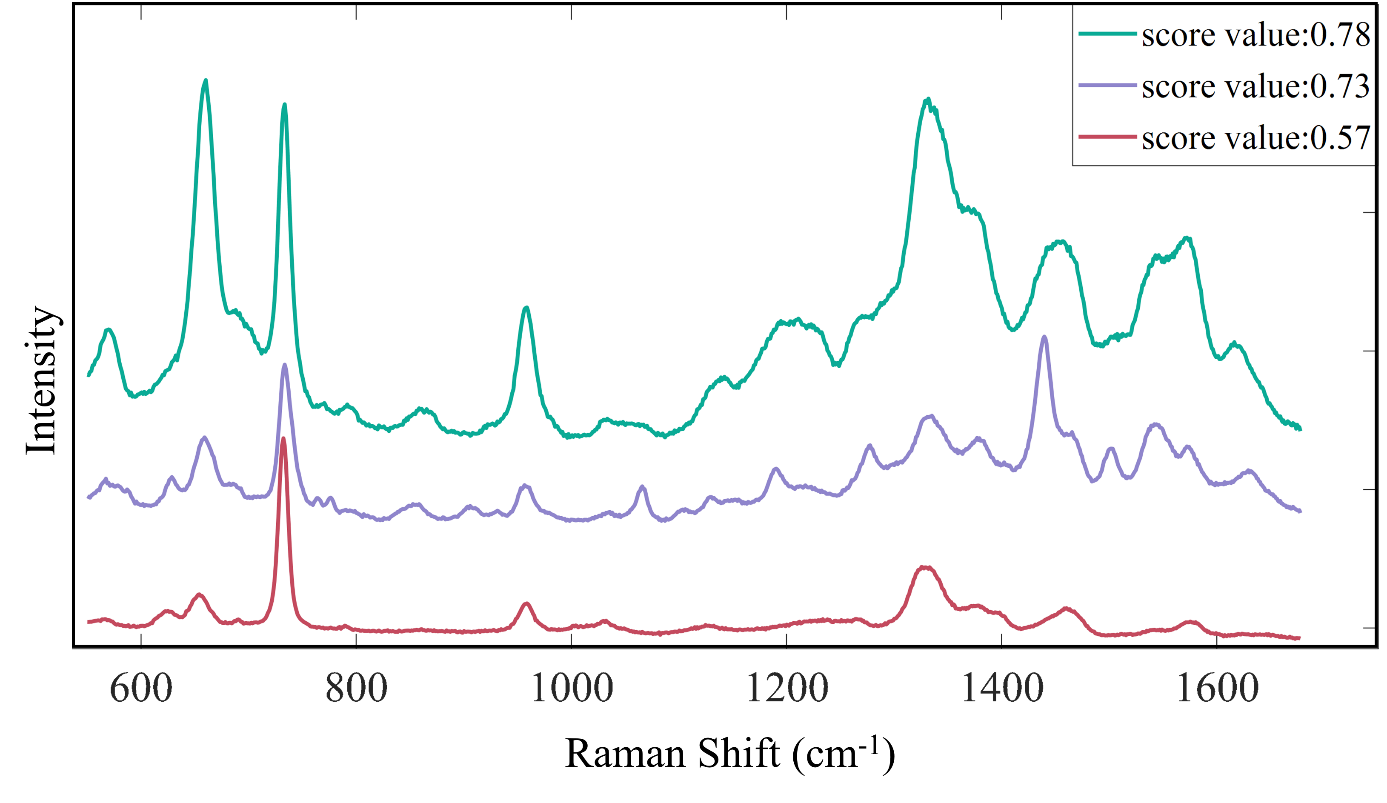


**Supplemental Figure S2.** Spectrum samples with different score values found in the isolation forest algorithm. Score values above 0.7 were accepted anomaly according to the Isolation Forest algorithm and discarded from the data set.
